# “One of those areas that people avoid” a qualitative study of implementation in miscarriage management

**DOI:** 10.1186/1472-6963-13-123

**Published:** 2013-04-03

**Authors:** Blair G Darney, Marcia R Weaver, Deborah VanDerhei, Nancy G Stevens, Sarah W Prager

**Affiliations:** 1 Departments of Medical Informatics and Clinical Epidemiology and Obstetrics and Gynecology, Oregon Health & Science University, 3181 SW Sam Jackson Park Rd, Mailcode L-466, Portland, OR, 97239, USA; 2 Department of Health Services, University of Washington, Seattle, WA, USA; 3 Global Health, University of Washington, Seattle, WA, USA; 4 Department of Family Medicine, University of Washington, Seattle, WA, USA; 5 Department Obstetrics and Gynecology, University of Washington, Seattle, WA, USA

**Keywords:** Miscarriage, Reproductive health services, Family medicine, Practice change, Interprofessional training, Dissemination, Process evaluation

## Abstract

**Background:**

Miscarriage is common and often managed by specialists in the operating room despite evidence that office-based manual vacuum aspiration (MVA) is safe, effective, and saves time and money. Family Medicine residents are not routinely trained to manage miscarriages using MVA, but have the potential to increase access to this procedure. This process evaluation sought to identify barriers and facilitators to implementation of office-based MVA for miscarriage in Family Medicine residency sites in Washington State.

**Methods:**

The Residency Training Initiative in Miscarriage Management (RTI-MM) is a theory-based, multidimensional practice change initiative. We used qualitative methods to identify barriers and facilitators to successful implementation of the RTI-MM.

**Results:**

Thirty-six RTI-MM participants completed an interview. We found that the common major barriers to implementation were low volume and a perception of miscarriage as emotional and/or like abortion, while the inclusion of support staff in training and effective champions facilitated successful implementation of MVA services.

**Conclusion:**

Perceived characteristics of the innovation that may conflict with cultural fit must be explicitly addressed in dissemination strategies and support staff should be included in practice change initiatives. Questions remain about how to best support champions and influence perceptions of the innovation. Our study findings contribute programmatically (to improve the RTI-MM), and to broader theoretical knowledge about practice change and implementation in health service delivery.

## Background

About 15% of recognized pregnancies end in miscarriage, or spontaneous abortion [1,2]; the proportion increases with the sensitivity of pregnancy diagnosis to a range of 20%-62% [3]. Miscarriage management strategies are expectant (wait and see), medication (misoprostol), office-based management via manual vacuum aspiration (MVA), and operating room based management under general anesthesia. MVA is as safe as operating room-based care in samples of women presenting with miscarriage [2] and seeking induced abortion [4,5], may improve patient satisfaction with care [1,6], and results in significant time and cost savings compared to operating room-based management [1,3,7]. However, operating room-based management has remained usual practice for decades. The literature suggests that non-complicated miscarriage cases (e.g. <12 weeks) should be counseled about the full range of management approaches [6]. Patient preferences have been shown to play a role in choice of management and in post-procedure satisfaction, [1,8,9] regardless of which management approach is chosen [9].

Family medicine residents are not routinely trained in office-based uterine aspiration for miscarriage [10] despite recommendations [11]. However, training family medicine residents to perform MVA in an office setting can improve continuity of care and expand access to this procedure, especially in rural settings served solely by a generalist [10,12]. Experience with MVA for miscarriage management is also a skill that can be translated to other common procedures: management of uterine hemorrhage, endometrial biopsies, and induced abortion.

The safety and efficacy of office-based uterine aspiration using MVA is known; less well understood is how to successfully integrate the service into family medicine settings. Our impact evaluation [13] showed a positive association with physician intent to practice MVA following a training intervention; this process evaluation sought to understand the implementation process—how MVA was or was not incorporated into routine practice—more comprehensively at participating sites.

**Figure 1:**
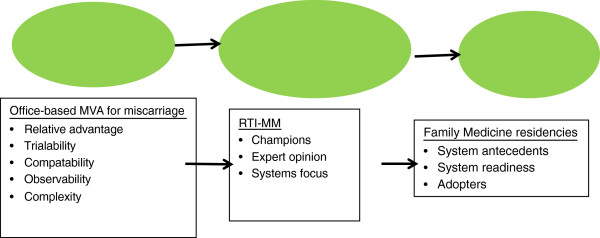
**Characteristics that support or impede practice change.** Adapted from Greenhalgh [14].

Multiple disciplines have focused on the concept of diffusion or implementation of new practices [14]; most trace the concept to Rogers’ work in rural sociology [15]. Definitions of diffusion, dissemination, and implementation vary, as does the conceptual focus of the literature. Some work has focused on different levels where implementation takes place (e.g. institutional, individual), while some focuses on the nuances of different patterns of diffusion or adaptations by users [16]. However, the central concern of this body of work is to describe how and why new practices are adopted. We selected a conceptual model that focuses on practice change in health service organizations, and is adapted from Greenhalgh et al.’s [14] unifying, cross-disciplinary conceptual model of the determinants of dissemination (active and planned efforts to persuade target groups to adopt an innovation) and implementation (active and planned efforts to mainstream an innovation within an organization) of innovations in health service organizations (Figure 1). This model explicitly acknowledges the role of the innovation, dissemination strategy, and the targeted or intended users, and incorporates both demand side (intended uses) and supply side (the innovation and dissemination strategies) characteristics [16]. The innovation in this project is office-based miscarriage management via MVA in family medicine residency settings, the Residency Training Initiative in Miscarriage Management (RTI-MM) is the dissemination strategy, and the user system organizations are the family medicine residency sites.

Dissemination strategies employed by the RTI-MM (described below) include many identified by Greenhalgh et al. [14] and others [15,17-21] as positively associated with successful implementation: interactive strategies and outreach visits, use of champions, expert opinion, tailoring, and an explicit focus on systems change. The RTI-MM also incorporated strategies to address characteristics of the innovation such as trialability and observability, and to influence perceptions such as relative advantage, compatibility, or cultural fit, complexity, and risk [14] which have been linked to diffusion [22] and active dissemination. The RTI-MM team had less information about the user systems, but hypothesized that residency programs would have good absorptive capacity for new knowledge [14], including a “learning organization culture,” and be open to interprofessional teamwork.

### RTI-MM program characteristics

The RTI-MM was aimed at family medicine residents, faculty, and clinical and administrative support staff at all 10 diverse, non-military family medicine residency sites in the State of Washington. The first step of the RTI-MM was to involve residency faculty as site “champions,” key individuals in the social network who support the innovation [15]. We held a workshop with representatives of all family medicine residency sites who indicated interest in promoting the success of the RTI-MM. Following the champions workshop, the training team traveled to each of the 10 residency sites. The standard RTI-MM on-site, half-day intervention package for faculty and residents included:

1. A didactic session on office-based miscarriage management (expectant, medication and MVA): *“Do nothing, do something, do surgery,”* with an explicit focus on patient-centered care (patient choice in miscarriage management options).

2. A hands-on simulation workshop to practice MVA technique. A ripe papaya makes a good uterine simulator. The papaya model has the advantages of being low-tech and relatively easy to implement, and permits participants to practice emptying the contents of the uterus (papaya seeds and fruit) with the MVA device, which plastic pelvic models do not [23].

3. Participatory discussion sessions focused on systems change, values clarification [24], patient centered care, and hesitations or perceived barriers to integrating office-based miscarriage management into routine practice.

Support staff (clinical and administrative) were encouraged to participate in the initial training described above and also received two additional on-site sessions tailored to their roles and clinical sites. Training content was tailored based on participant feedback at the stakeholder meeting and trainer experience during the initial didactic session. Support staff training typically included basic didactic information on miscarriage management in the office setting and focused on systems change to address site-specific barriers such as patient flow, triage, billing, and blood products management. All 10 sites received the standard didactic and two support staff trainings.

## Methods

This process evaluation is part of a mixed-methods evaluation of the RTI-MM [13]. In this study, we used purposive sampling and qualitative methods to identify barriers and facilitators to successful implementation of miscarriage management services using MVA.

Data collection took place between 6 and 18 months after the initial training session and after all training sessions were completed; timelines were different for each site based on project roll-out. All individuals who attended an RTI-MM training session received a recruitment email from the Family Medicine Residency Network (FMRN), a co-coordinating body of family medicine residency sites. Potential participants contacted the evaluator (BGD) to learn more about the study, review the consent process, and schedule an interview. All interviews took place after receipt of written informed consent and were conducted over the phone to provide participants with maximum flexibility in scheduling. Phone interviews were recorded as password-protected electronic computer files. This study was approved by the University of Washington Human Subjects Division.

We employed purposive, maximum variation sampling [25] (p. 243). We oversampled at two of the 10 sites (two sites had been selected to participate in additional follow-up training after data collection for this study was complete). Semi-structured interviews followed a general interview guide approach [25] (p. 343). That is, the interview guide contained all possible questions, but not all participants were asked the same questions in the same order; flexibility was built into the data collection process. Interviews ranged from 15–75 minutes (average 25 minutes). Participants were prompted during the interview to answer questions about whether they had used MVA prior to the training, if their site had implemented the service, major barriers to implementation, how the site champion functioned at their site, and whether the similarity of miscarriage management and abortion care was a challenge to implementation at their site.

The first author (BGD) transcribed each interview into a case summary organized by interview question as a first stage of data reduction and synthesis. She then read all transcripts, noting the emergence of overarching themes. This first version of the code scheme was broad, spanning many topics that reflected the theoretical focus of the guiding conceptual model (e.g. grouped by the innovation, dissemination strategies and the user system). After consultation with co-authors, a second read focused on selecting five case summaries that best represented the breadth of the data to code using Atlas.ti qualitative data management software (Atlas.ti Scientific Software Development, GmbH, Berlin, Germany). Coding was an iterative process. A short initial code list was developed; next, we refined the code list to include emergent themes [26]. Finally, after all case summaries were coded, we refined the code list a final time, merging overlapping codes and renaming codes. For example, we decided to collapse codes about miscarriage and death into the larger code of miscarriage is emotional. We were not interested in miscarriage and death specifically, but were interested in death as part of the perception of miscarriage as emotional that posed a barrier to implementation. Following data coding, we developed matrices [27] to display summarized data by key themes across and within subjects [28] and stratified by role and site [25] to facilitate comparative analyses [29] and examination of themes in the context of our conceptual framework.

## Results

Thirty-six RTI-MM participants completed an interview (Table 1). The 10 sites contributed a range of one to 10 interview participants, with a modal value of three. Miscarriage management varied at the 10 sites prior to the training. No sites offered office-based aspiration miscarriage management services using MVA prior to the RTI-MM training, but half offered medication management and all did expectant management. Prior to the RTI-MM, all patients selecting aspiration management were referred out to be managed in an operating room setting. At the time of interviews, 6 of the 10 sites had implemented MVA services; 3 additional sites were eventually able to implement but had not at the time of interviews. Implementation meant that the site had all necessary equipment and protocols in place and had managed at least 1 case using the MVA. Twenty-six of the 36 interview participants came from sites that had implemented MVA. Table 1 describes interview participant characteristics. We present our results classified by the conceptual framework as related to the innovation, the dissemination strategy, and the user systems.

**Table 1:** Participant characteristics, N=36

	***n***
Role	
Resident	10
Faculty	12
Clinical support staff (e.g. MA, RN)	8
Administrative support staff (e.g. clinical manager, scheduler)	6
Site champions (MD or support staff)	7
Female gender	27
White, non-Hispanic race/ethnicity (n = 5 missing)	26
At a site that implemented MVA services by the time of interviews (n=6 sites)	28
At a site that was selected for follow-up training following this study (n=2 sites)	17
At a site that provides induced abortion services (n=2 sites)	5
At a site that provides any induced abortion training (including off-site opt-in training; n=3 sites)	8

### The innovation: perceptions of miscarriage and MVA

Perceptions of miscarriage and of the MVA device or procedure were barriers to implementation of MVA services for miscarriage. Miscarriage was perceived as emotional and as like induced abortion; achieving clarity on the difference between miscarriage and abortion was important to user systems and facilitated implementation. Low volume and thus lack of opportunity to train using the MVA was also a barrier for sites.

#### It is a very emotional sort of thing

Participants focused on perceptions of the diagnosis and treatment of miscarriage as highly emotional and thus a barrier to implementing the service.

“…And this is an area that people, you know, it’s like I don’t want to deal with this it’s bloody and it’s emotional and get me out of here. And their default was just to send them somewhere else so they didn’t have to deal with it.” (Faculty MD and site champion)

Some of the emotional nature of miscarriage was described as linked to providers’ circumstances and discomfort, not patient emotions.

“I guess, you know, it’s hard for [staff] to talk about, it’s the what if, it’s the death concept, it’s just one of those areas that people kind of go all around to avoid sometimes.” (Support Staff, LPN team leader)

#### It’s different but it’s done like an abortion

Much of the perception of miscarriage as emotional stemmed from the proximity of miscarriage and induced abortion services. Participants spoke about how miscarriage management was like abortion while also clearly articulating the difference. In addition, resistance to MVA services due to concerns about abortion often originated with support staff. 

“…the idea that this procedure started with elective abortions…and it was now coming over to miscarriage management [and] just being able to emotionally disconnect those two because I have such strong feelings about abortion.” (Support staff, Nurse manager)

Others described how the MVA equipment or procedure itself was a barrier for support staff: 

“The [RTI-MM was] enriching of training we already did for support staff [who identify as anti-abortion], they were able to think about the procedure itself in a more positive light.” (Faculty MD and site champion)

#### Knowing the distinction and how to tell the difference

Achieving clarity on the difference between miscarriage management and abortion services during the RTI-MM training sessions facilitated implementation.

“ Many of the people that I talked with…[said] we’re not doing an abortion are we? So once they understood where in the process patients would be, they realized we were not doing abortion…then they were happy to be supportive and be a part of it.” (Support staff, Nurse clinic manager)

Some participants, mostly but not exclusively physicians, self-identified during interviews as pro-choice. These participants had a different perspective on the similarity of abortion and miscarriage services. Some expressed that focusing on emotions or abortion either in training or within a site was problematic. 

**Table 2:** Additional quotes grouped by theme

	
**The innovation: perceptions of miscarriage and MVA**	
*“It is a very emotional sort of thing”*	“For residents and for trainees it’s the emotional content and sort of the technical content and trying to manage them both at once…In other procedures that we’ve introduced in recent years, the emotional content isn’t as high. I think it’s hard for people to balance that when you’re working so hard to learn a technical skill, then it’s hard to learn about caring for the patient at the same time, balancing those two things is really hard, but it’s what you have to learn.” (MD faculty and site champion)
*“Knowing the distinction and how to tell the difference”*	“And then having in the miscarriage management [training] a whole lot of talk about the procedure being upsetting and all that I think it really emphasized the connection of miscarriage management to therapeutic abortion instead of normalizing the procedure.” (Faculty MD and site champion).
*“Every time it's the first time.”*	“I think one of the big challenges is that it’s so rarely needed that every time you do you’re reinventing the wheel. And although we have a pretty good volume, we don’t have the volume that one would need to do enough procedures to make everybody feel comfortable.” (Faculty MD and site champion)
*“Its pretty simple”*	“Being able to do this in our clinic is a cost saver for patients…our patients are more likely to follow through and I think patients appreciate the procedure …” (Faculty MD)
**The RTI-MM dissemination strategy: champions, hands-on practice, and team training**	
*“Ideas how other clinics rolled out the service”*	“I mean I had MVAs and stuff, but we hadn’t quite gotten to the point of having protocols and using them in the clinic” (Faculty MD and site champion)
**The user systems: teamwork and scope of practice**	
*“Hearing the same messages”*	“I’m kind of the…resource person for so much of what happens in the clinic. So although I might not ever be involved in one of the procedures (although I’d like to be just so I can be more tuned into what happens), I definitely need to know what’s going on.” (Support staff, Nurse supervisor)
*Scope of practice…is a battle here*	“it’s a little bit political…we still end being a little more on the defensive and that’s part of the problem…as opposed to if I was a rural doc and I was the only one who offered this procedure, then everybody would be delighted I did it and nobody would give me a hard time about it, but in the urban setting, when there’s maybe a little bit different community standard and our OB backup isn’t doing miscarriage management in the clinic setting, then that feels a little bit trickier.” (Faculty MD)

For some a connection between miscarriage and abortion services was positive:

“I think we have to acknowledge that this is a way of teaching abortion skills to family medicine doctors who otherwise don’t get this in clinics where abortion is not provided, right.” (Faculty MD)

#### Every time it’s the first time

Low case volume of miscarriage patients in general and of patients who choose MVA in particular emerged as a major perceived barrier to implementation.

“If we don’t do something regularly, it makes people nervous when it does come up.” (Support staff, MA)

#### It’s pretty simple

In contrast, some attributes of MVA for miscarriage management were identified by participants as facilitators to implementation: that it was easy to use, would lower patient and health care system costs, or could improve quality and or continuity of care.

“I think people appreciate[d]…how simple a procedure and how quick it is, and…[patients] can avoid anesthesia, they can do it right there in my office, I can schedule at the end of the day and just get it over with, I do think there’s value in that.” (Resident MD).

Speaking about a miscarriage case managed using MVA, one participant talked about how the MVA permitted better continuity of care: 

“She was able to have her husband in the room, she had her regular physician, she had the interpreter in the room whom she knew, I think it was a very comfortable mentally for her to have it done in our clinic in familiar surroundings.” (Support Staff, LPN)

### The RTI-MM dissemination strategy: champions, hands-on practice, and team training

Champions—what they do and how to support them—were central to implementation of MVA services. In addition, a positive or negative initial user system experience with MVA, often managed by the champion, was important to implementation. Other components of the RTI-MM dissemination strategy cited as helpful to implementation were the hands-on papaya workshop, inclusion of support staff in training, and a focus on clinic systems.

#### More of a reminder that it is an option

Champions and other participants described what champions did or did not do, and the challenges they faced. One faculty member provided a detailed description of the role of the champion at her site:

“[the champion] said this is important, we need to start doing this, you can’t wait until you have a perfect protocol and the perfect patient walks in to start doing things, or you’ll never do anything…and I think [she] was right, if we could start doing this and be successful, then it wouldn’t feel so daunting…she kept bringing it up and that kind of got us over that initial hump. And it does really need someone to champion it in an ongoing fashion…who really says this is really good, is anybody thinking about this for these patients, how has this gone, is there anything we can do to make it go better, check in with the nurse…sort of how can we make sure that everyone feels like this is a success.” (Faculty MD)

Champions provided ongoing reminders about the innovation, momentum, and established new norms; they also worked to ensure that initial experience with the MVA was as positive as possible to promote a smooth and successful experience for the team.

Strong champions also commanded respect from peers. Support staff who took on a champion role could be especially effective: 

“…but most importantly one of our LPN champions was very positive about it…he has a lot of respect from the MAs and the staff.” (Faculty MD and site champion)

Champions faced many challenges such as competing priorities and unsupportive clinic or institutional environments. The challenge of championing alone was cited by several participants:

“we have you know one doctor…this has really been [in] her heart to…get miscarriage management here…she is amazing, [but] she’s involved in so many other things,” (Support staff, LPN team leader)

One participant offered advice on how to support champions using expert or peer advice: 

“When somebody feels like they are championing alone, having someone…outside of the clinic, who says ‘we do this, we’ve been successful doing this, how else can we help you?’ would be great.” (Faculty MD)

#### Just getting comfortable…which buttons to push and just getting used to it

Many participants cited the hand-on simulation exercise with the papaya model as the most beneficial part of the training because it allowed them to practice aspiration technique in a realistic way. 

“The papaya it’s nice for people to get their hands on the syringe and just get a sense of what the suction feels like. It’s nice to have something appear in the syringe and I think gave people a little more confidence…” (Faculty MD and site champion)

#### Ideas how other clinics rolled out the service

Participants spoke about other aspects of dissemination focused on their systems such as protocols, patient education materials, and hearing how other sites had implemented MVA. This content was necessary for implementation, and also often necessary to convince organizational leaders to endorse the practice change initiative. 

“[During the RTI-MM,] we discussed scenarios and options and questions and got some ideas how other clinics rolled out the service and used it and we got some [protocols, education materials]…that we could incorporate into our practice.” (Support staff, LPN office co-ordinator).

### The user systems: teamwork and scope of practice

Each residency site was a unique user system, but common themes emerged, especially about the value of team, or interprofessional, training and scope of practice.

#### Hearing the same messages

The inclusion of support staff in didactic training and focus on values and systems within the support staff training were cited as unique and useful characteristics of the RTI-MM. Participants acknowledged that support staff involvement is necessary to successful implementation: 

“critical to having it happen is…support staff who want to make it happen.” (Resident MD)

Support staff also spoke of their role in service provision and resident training:

“interesting…since we are in a teaching situation to have the hands on experience with the papaya and you know be able to really see what the doctors are doing because that helps us in training with the doctors.” (Support staff, MA)

#### Scope of practice for family practitioners is a battle here

Perceptions of scope of practice within each site varied and depended on several factors including relationships with obstetrician-gynecologist colleagues, patient population, and geographic location. However, these factors were not deterministic and operated differently across sites, indicating that local culture is central to scope of practice controversies. Obstetrician-gynecologists were perceived as having influence over family medicine clinic policies:

“I’ve talked with him [staff Ob/Gyn] about it and his bias is that patients have a preference to just do it in the OR rather than doing in the clinic. So that could be it too, because I imagine he has an influential role around our clinic policies and that sort of thing.” (Resident MD)

Scope of practice was also perceived to be a potential issue at the organizational or hospital level: “[I was] unsure if the [hospital] powers that be…would be OK with us providing that service or if it would be deemed oh, that’s only something that OB/Gyns can do.” (Resident MD)

For each key theme we have presented supporting data; additional data to illustrate themes are in Table 2.

## Discussion

Our data about the implementation of MVA for miscarriage management in Family Medicine residency settings in Washington State support the importance of previously identified constructs in the implementation process [14,30]. We found that perceptions of the innovation, the dissemination strategies, and the user system all played a role in the implementation process. We identified common barriers to implementation such as low volume and a perception of miscarriage as emotional and/or like abortion, as well as facilitators, such as the inclusion of support staff in training, hands on experience, and effective champions. Our findings also raise questions for further study, such as the best way to support champions and the interaction between champions and their systems, and identification of the best strategies to positively influence perceptions of the MVA procedure and miscarriage.

### The innovation

Our results strongly support previous work that states that user perceptions of the innovation are central to implementation [14,15,22,30]. This concept considers the “goodness of fit” between an innovation and the adopters [16]. In our sample, perceived characteristics of the innovation were about both the device and procedure (MVA) and about the diagnosis (miscarriage). No practice change initiative is neutral, but miscarriage, with its proximity to death, reproduction, and induced abortion, may be especially value-laden and therefore challenging. Perceived characteristics of the innovation (like abortion, too emotional) and perceived compatibility of the innovation with individual users and systems due to these perceptions must be explicitly addressed in dissemination strategies. There was overlap and feedback between the innovation and users and their systems, highlighting that these categories or constructs do not have clear boundaries [30]. For example, perceptions of miscarriage and thus the MVA procedure as “too emotional” speak to the cultural characteristics of the innovation (MVA) and also to the beliefs and preferences of the user system [16].

Previous literature suggests that individuals or sites with a focus on patient centered care are more successful at implementing innovations [30]. We found that patient centered care, especially the concept of continuity of care, was a value shared by users that enhanced compatibility and facilitated implementation of MVA, and that for some users, the MVA itself had attributes that enhanced patient centered care – simple, quick to use, and inexpensive.

### Dissemination strategies

It is well understood that compelling evidence is necessary but not sufficient for practice change to occur; evidence must be supported with additional strategies [17,18,22,31]. The use of champions, interactive approaches, and a systems approach to team training were all important to the RTI-MM dissemination strategy. Our results point to the importance of effective champions and provide detail about how champions operate. Effective champions were those leaders who maintained a focus on integrating MVA into practice, who engaged in training the entire team, who leveraged existing networks, such as support staff leaders, and who were able to encourage change in clinic norms or expectations. Strong champions were able to address technical aspects of implementation as well as cultural or political “fit” within their organizations [16]. Champions addressed lack of cultural fit due to perceptions of the MVA procedure or miscarriage by setting new norms; this is a type of conformity pressure, recognized as a mechanism to affect lack of cultural fit [16]. Experience—a positive or negative experience with MVA and how that feeds back into implementation—was a key role of champions at participating sites. Reliance on a single site champion can lead to burnout, however, given that champions have many competing responsibilities and priorities [32]. Our experience suggests that supporting champions is a key component of success and our findings point to ways to develop and support effective champions, such as providing content for protocols and presentations to colleagues or hospital administration about MVA and checking in frequently to share new information and provide support.

Our results support using values clarification in practice change interventions to address barriers related to value-laden perceptions about the innovation. Values clarification has been widely used as part of patient decision-making tools [33,34], but used less frequently to examine provider behavior. A key exception to this is in the literature on implementing induced abortion services [24,35,36]. Future research should explore whether exposure to values clarification shifts physician and support staff attitudes about miscarriage and whether exposure to miscarriage can shift attitudes about the MVA procedure or induced abortion services.

### The user system

The inclusion of clinical and administrative support staff in RTI-MM training facilitated implementation of MVA services. The RTI-MM explicitly acknowledged the important roles of support staff in patient care and in working with faculty to train residents. Even in these teaching sites, training did not routinely include support staff, and some sites were not initially enthusiastic about the inclusion of support staff in training. Interprofessional and/or team training is an innovation in medical [37,38] and continuing [39] education, facilitates implementation [40], and has been associated with better clinical preparedness by physicians [41]. However, it is challenging to implement [42] and does not yet have strong evidence to support impacts on professional practice or health outcomes [43-45]. User systems that successfully implemented MVA were generally those sites where study participants articulated their role as learning and training centers, acknowledged the role of support staff in implementation and patient care, and “bought into” the team (interprofessional) training component of the RTI-MM.The role of interprofessional education in reproductive health services implementation deserves further study.

Our results should be interpreted with the following limitations in mind. Our results may not be generalizable beyond Washington State; there may be differences in residency education and perceived scope of practice across states. We oversampled at sites selected for further follow-up, which may limit generalizability to all sites. However, our findings across sites support findings at our oversampled sites and our use of maximum variation sampling ensured that a variety of roles and sites provided data. As with all non-experimental research designs, it is likely that study participants had more interest in our topic than non-participants; it is also possible that non-participants experienced different barriers and facilitators than study participants. However, our data represent a range of opinion about implementation of miscarriage management services. Finally, we collected data from individuals and look for common themes at the site level. Sites are made up of individuals, and organizational change begins with individuals, but we do not understand how they interact [30].

## Conclusion

Our study of implementation of miscarriage management services using MVA provides concrete strategies for clinical sites seeking to successfully implement similar services. We found much that overlaps with the broader health services, practice change, and implementation science literature, and also identified elements that may be specific to reproductive health care services. Perceived characteristics of the innovation and perceived compatibility, or fit, of the innovation with individual users and systems must be explicitly addressed in dissemination strategies; supporting champions is a key component of success; and support staff should be explicitly included in practice change interventions. While these components support existing literature, perceived characteristics of the innovation may be a larger barrier in reproductive health services than other primary care services. Questions remain about how to best support champions and influence perceptions of the innovation, and which components of the RTI-MM dissemination strategy were most important. Our study findings contribute programmatically, to improve the RTI-MM, and to broader theoretical knowledge about practice change and the implementation of innovations in health service delivery.

## Competing interests

The authors declare that they have no competing interests.

## Authors’ contributions

BGD designed the study, collected the data, lead the analysis, and drafted the manuscript. MRW assisted with study design, conceptual framework, analysis, and manuscript writing. DV assisted with analysis and interpreting results. NGS assisted with study design, participant recruitment, and interpreting results. SWP assisted with interpretation of results and manuscript writing. All authors read and approved the final manuscript.

## Pre-publication history

The pre-publication history for this paper can be accessed here:

http://www.biomedcentral.com/1472-6963/13/123/prepub
